# Circulating microRNAs: a novel potential biomarker for diagnosing acute aortic dissection

**DOI:** 10.1038/s41598-017-13104-w

**Published:** 2017-10-06

**Authors:** Jian Dong, Junmin Bao, Rui Feng, Zhiqing Zhao, Qingsheng Lu, Guokun Wang, Haiyan Li, Dingfeng Su, Jian Zhou, Qing Jing, Zaiping Jing

**Affiliations:** 1 Department of Vascular Surgery, Changhai Hospital, Second Military Medical University, Shanghai, China; 2 Department of Surgery, Changhai Hospital, Second Military Medical University, Shanghai, China; 3 Department of Cardiology, Changhai Hospital, Second Military Medical University, Shanghai, China; 4 Key Laboratory of Stem Cell Biology and Laboratory of Nucleic Acid and Molecular Medicine, Institute of Health Sciences, Shanghai Institutes for Biological Sciences, Chinese Academy of Sciences & Shanghai Jiao-Tong University School of Medicine, Shanghai, China; 5 Department of Pharmacology, Second Military Medical University, Shanghai, 200433 China

## Abstract

Acute aortic dissection (AAD) is a catastrophic emergency with high mortality and misdiagnosis rate. We aimed to determine whether circulating microRNAs allow to distinguish AAD from healthy controls and chest pain patients without AAD (CP). Plasma microRNAs expression were determined in 103 participants, including 37 AAD patients, 26 chronic aortic dissection patients, 17 healthy volunteers, 23 patients without AAD. We selected 16 microRNAs from microarray screening as candidates for further testing via qRT-PCR. The results showed that plasma miR-15a in patients with AAD (n = 37) had significantly higher expression levels than it from control group (n = 40; *P* = 0.008). By receiver operating characteristic curve analysis, the sensitivity was 75.7%; the specificity was 82.5%; and the AUC was 0.761 for detection of AAD. Furthermore, 37 patients with AAD had significantly higher plasma expression levels of let-7b, miR-15a, miR-23a and hcmv-miR-US33-5p compared with 14 CP patients of 40 controls (*P* = 0.000, 0.000, 0.026 and 0.011, respectively). The corresponding sensitivity were 79.4%, 75.7%, 91.9% and 73.5%, respectively; the specificity were 92.9%, 100%, 85.7% and 85.7%, respectively; and the AUCs of these microRNAs were 0.887, 0.855, 0.925 and 0.815, respectively. These data indicate that plasma miR-15a and miR-23a have promising clinical value in diagnosing AAD.

## Introduction

Acute aortic dissection (AAD) is one of the most catastrophic cardiovascular diseases with high mortality^1,2^, while diagnosis and treatment of AAD have been greatly improved in recent years, including noninvasive imaging diagnosis^3^ and endovascular treatment from descending aorta to ascending aorta^4–6^. An early and correct diagnosis may warrant immediate initiation of effective therapy to potentially reduce the mortality rate^7^. Current diagnosis of AAD requires definitive imaging such as computed tomography, magnetic resonance imaging, or transesophageal echocardiography, but the use of each investigation is based on an index of clinical suspicion, and each incurs a further logistical delay in patient management^8^. Diagnosis is therefore not immediate, and the varied presentation allows the diagnosis to be missed, misdiagnosed, or overlooked in up to 39% of cases^9^, sometimes only being established at post-mortem^10^.

A biomarker has great potential to improve the early diagnosis of AAD^11^. There are a number of candidate biomarkers, including smooth muscle myosin heavy chain, calponin, soluble elastin fragments and D-dimer which were shown to be useful within a short time-window or without thrombosis of the case^11–14^. Another biomarker with higher sensitivity and specificity for diagnosis of AAD that can extend the time-window to later stages would thus be expected.

MicroRNAs are a set of small non-coding RNAs, with the length of 18 to 22 nucleotide^15,16^, several of which have been reported to play an important role in cardiovascular development and diseases^17–19^. We have demonstrated that miR-30 might play important roles in the pathogenesis of AAD by regulation of the mitogen-activated protein kinase pathway^20^. Recent studies have shown that the levels of some circulating microRNAs were associated with the diagnosis and prognosis for diseases^21^. Our previous study demonstrated that plasma miR-208a might be a novel biomarker for acute myocardial infarction^22^. In this study, we investigated the possibility of plasma microRNAs as biomarkers for AAD diagnosis.

## Methods

### Population

63 aortic dissection (AD) patients (37 AAD and 26 subacute aortic dissection [SAD]), and 23 non-aortic dissection (non-AD) patients admitted to the Department of Vascular Surgery in Shanghai Changhai Hospital between February 2011 and July 2012 were recruited in the study. The diagnosis of AD was based on computed tomography angiography with contrast confirming a dissected aorta containing both a true and false lumen. The non-AD patients include 11 patients with acute myocardial infarction (AMI), 10 patients with aortic aneurysm (AA) and 2 patients with pulmonary embolism (PE). In addition, 17 healthy volunteers (without cardiovascular disease history, 15 [88.2%] males, [55.0 ± 5.3] years) were enrolled in this study. The control groups included all participants without aortic dissection (n = 40). Among them, 14 participants have symptoms of chest pain (CP), including 11 AMI patients, 2 PE patients and 1 AA patient.

The protocol of this study was carried out according to the principles of the Declaration of Helsinki and approved by the Medical Ethics Committee in Shanghai Changhai Hospital. Written informed consent was obtained from all the participants before enrollment.

### Plasma Collection and Storage

Blood samples for microRNAs detection were collected from patients in the emergency department or the vascular surgery laboratory and were processed within 1 hour of collection by two-step centrifugation. The supernatant was transferred to RNase/DNase-free tubes and stored at −80 °C.

### RNA Isolation

Total RNA were isolated from plasma specimens using miRNeasy Mini Kit (Qiagen, Hilden, Germany) following the instructions from the manufacturer. The Caenorhabditis elegans microRNA (cel-miR-39) was synthesized for the spiked-in control. 0.4 pmol cel-miR-39 was supplemented into each 200 ul sample, which had been mixed with 1.0 ml QIAzol lysis Reagent (Qiagen, Hilden, Germany). The quality of total RNA samples was detected by NanoDrop ND-2000 Spectrophotometer (Thermo Scientific Wilmington, DE, USA). Only the samples with A260/280 > 2.0 and A260/230 > 1.8 were used for further analyses.

### Microarray and qRT-PCR

Human MicroRNA Microarrays (Release 16.0, 8 × 60k) (Agilent Technologies, Santa Clara, CA) were used to identify candidate microRNAs. The Complete Labeling and Hyb Kit (Agilent Technologies, Santa Clara, CA, US) was used to label the miRANs samples, and hybridized with Cy3-tagged RNA. Then the quantization of the image information was performed with Feature Extraction Software 10.7. Quantile algorithm (Gene Spring Software 12.6) was applied to normalize the raw data. The samples with intra-array coefficients of variation (CV) above 15% would be discarded. The prediction analysis of microarrays (PAM) was used for microarray analysis. All microarray data has been submitted to GEO (GSE 92427).

For testing of candidate microRNAs acquired on microarray, quantitative reverse transcriptase-polymerase chain reaction (qRT-PCR) was performed. The RNA sample with high quality was used for reverse transcribed reaction which was carried out by the TaqMan miRNA Reverse Transcription Kit (Applied BioSystems, Foster City, CA). Then qRT-PCR reaction was performed using Taqman microRNA assays (Applied Biosystems, Foster City, CA) in a ABI PRISM 7900HT Sequence Detection System (Applied Biosystems, Foster City, Calif., U.S.A.). The total volume of the reaction was 10 μL, containing 5 μL of Universal Master Mix (Applied Biosystems, Foster City, Calif., U.S.A.), 2 μL cDNA template, and 3 μL. water. The primers used in the study were designed according to the sequences of the candidate miRNAs. The amplification condition was as follows: 95 °C template denaturation for 10 min, followed by 40 cycles at 95 °C for 15 seconds and 60 °C for 1 minute. U6 served as an internal control. Each reaction was repeated in triplicate. The data were analyzed with an automatic setting for assigning baseline; the threshold cycle was defined as the fractional cycle number at which the fluorescence exceeds the given threshold. The microRNAs which showed CT values above 40 cycles in greater than 20% of the 103 was considered not pass the quality control. The plasma levels of microRNAs were detected and analyzed by two investigators who were blinded to the clinical data of patients. The data obtained by real-time PCR were translated in log2 (relative level).

### Statistical Analysis

The prediction analysis of microarrays (PAM) was used for microarray analysis. The quantitative data were evaluated whether they followed the normal distribution by the Shapiro-Wilk test. The basis for declaring a certain parameter as normally distribution was *P* = 0.20. For the data that did not fit the normal distribution, the Kruskal-Wallis test was performed, while Levene’s test of homogeneity of variance was used for the analysis of data with normal distribution,. When the data fit the homogeneity of variance, one-way analysis of variance was applied, and for the data that did not fit the homogeneity of variance, the Kruskal-Wallis test was performed. The qualitative data were compared with Fisher’s exact test. The predicted probability of being diagnosed with AAD was used as a surrogate marker to construct receiver operating characteristic (ROC) curves. The area under the ROC curve (AUC) was used as an accuracy index for evaluating the diagnostic performance of the selected microRNAs. All *P*-values were two-sided, and any value less than 0.05 was considered statistically significant.

## Results

### Characteristics of Study Population

The characteristics of the study subjects were presented in Table 1. There were no statistically significant differences between AAD patients and controls (healthy volunteers and non-AD patients) for any of the considered variables (*P* > 0.05 for all).

**Table 1:** Characteristics of the Subjects.

Variable	Microarray					Real-Time PCR					
	AAD(n = 8)	Control Groups		*P*_1_	*P*_2_	AAD(n = 37)	SAD(n = 26)	Control Group (n = 40)	CP Group (n = 14)	*P*_3_	*P*_4_
		Healthy Controls(n = 8)	AA(n = 8)								
Age, mean ± SD, y	52.9 ± 16.6	53.8 ± 5.3	60.3 ± 6.2	0.517	0.340	57.7 ± 13.1	53.3 ± 11.0	57.3 ± 6.7	56.6 ± 7.3	0.875	0.717
Sex				1.000	0.352					1.000	1.000
Male, n (%)	8(100.0)	7(87.5)	8(100.0)			33(89.2)	25(96.2)	36(90.0)	13(92.0)		
Female, n (%)	0(0)	1(12.5)	0(0)			4(10.8)	1(3.8)	4(10.0)	1(7.1)		
Current smoking, n (%)	4(50.0)	2(25.0)	4(50.0)	0.673	0.504	12(32.4)	8(30.8)	17(42.5)	7(50.0)	0.362	0.334
DM, n (%)	1(12.5)	0(0)	0(0)	0.333	0.352	4(10.8)	0(0)	5(12.5)	3(21.4)	1.000	0.376
Hypertension, n (%)	8(100)	8(100)	7(87.5)	1.000	0.352	33(89.2)	16(61.5)	30(75.0)	10(71.4)	0.143	0.192
Hyperlipidemia, n (%)	0(0)	1(0)	0(0)	1.000	0.352	7(18.9)	4(15.4)	7(17.5)	4(28.6)	0.872	0.467
Marfan syndrome, n (%)	0(0)	0(0)	0(0)	1.000	1.000	0(0)	0	0(0)	0(0)	1.000	1.000

AAD, acute aortic dissection; SAD, subacute aortic dissection; AA, aortic aneurysm; DM, diabetes mellitus; CP, chest pain. Health is defined as not suffering from major diseases requiring hospitalization. *P*_1_: comparison between patients with AAD and control groups in screening. *P*_2_: comparison among patients with AAD, with AA and healthy controls. *P*_3_: comparison between patients with AAD and control groups in validating. *P*_4_: comparison between patients with AAD and CP groups in validating.

### MicroRNAs Screening and Training

**Figure 1:**
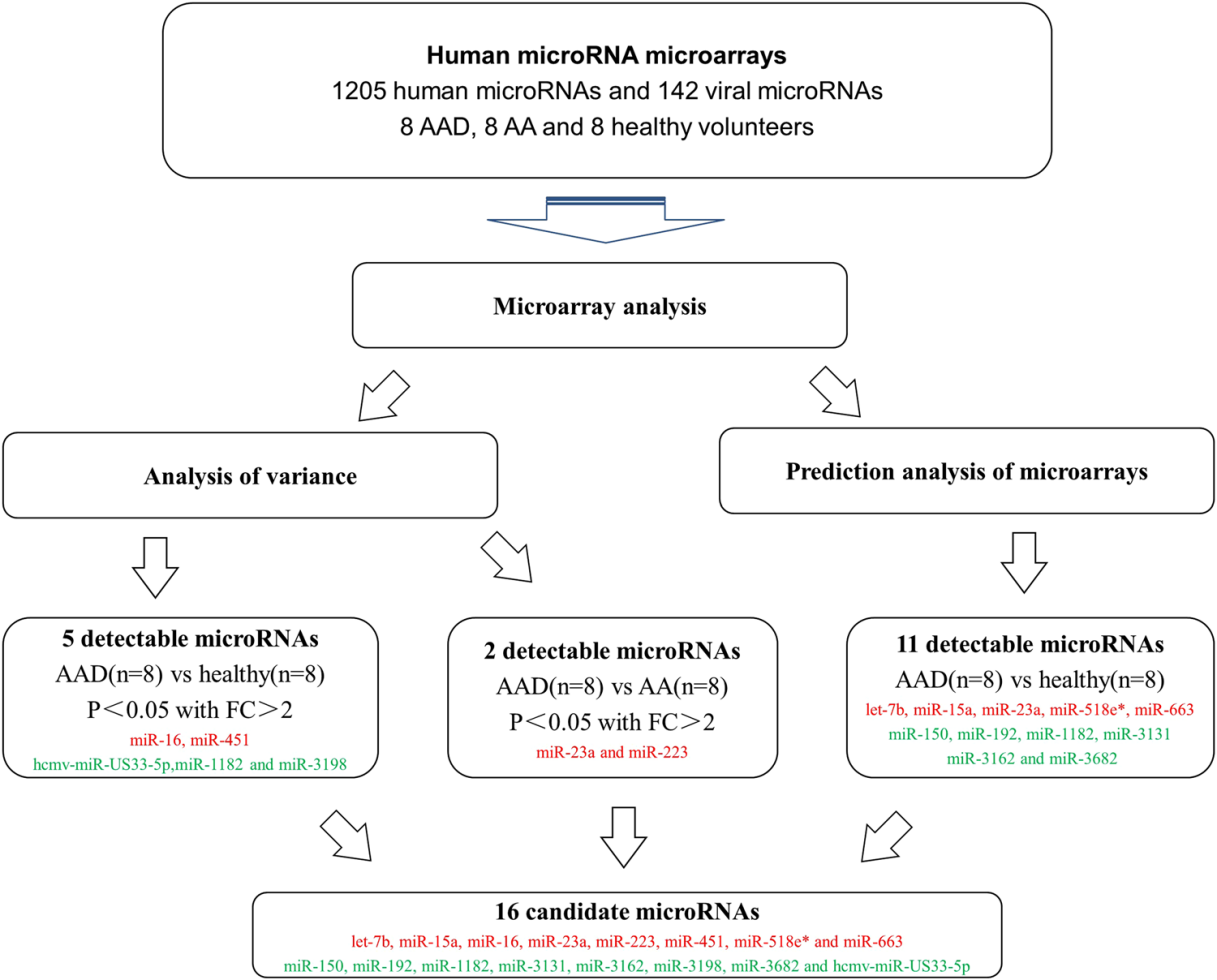
Process for Selection of Candidate MicroRNAs. Microarrays with 1,205 human microRNAs and 142 viral microRNAs were used for screening candidate diagnostic markers in the 3 categories of subjects from 24 plasma samples including AAD, healthy and AA subjects. There were two microRNAs overlapping in the 3 group comparisons. Finally, 16 candidate microRNAs discovered via microarrays were selected for the further validation. AAD, acute aortic dissection; AA, aortic aneurysm.The red flag indicates higher expression, and the green flag indicates lower expression.

A microarray containing probes for 1,205 human microRNAs and 142 viral microRNAs was initially used to screen the significant differential expression levels of microRNAs between the AAD (n = 8) and control groups (n = 16, 8 aortic aneurysm patients and 8 healthy volunteers). As shown in Fig. 1, the results illustrated the hierarchical clustering of the differentially expressed microRNAs in the pairwise comparison of the AAD and healthy groups, and AAD and aortic aneurysm, respectively. There were seven microRNAs, including let-7b, miR-15a, miR-16, miR-23a, miR-451, miR-518e* and miR-663, with significantly higher expression levels in the AAD group than in the healthy group. In contrast, miR-150, miR-192, miR-1182, miR-3131, miR-3162, miR-3196, miR-3682 and hcmv-miR-US33-5p exhibited significantly lower expression levels in the AAD group than that in the healthy group. When compared with patients with aortic aneurysm, patients with AAD showed up-regulated expression of miR-23a and miR-223. In summary, 16 abnormally expressed microRNAs were identified as candidates for further testing via qRT-PCR.

The 16 candidate microRNAs were tested using the entire sample set (63 AD and 40 without AD) with qRT-PCR, and 8 of the 16 microRNAs passed quality control. Among the miRNAs, miR-15a had a significantly different pattern between the AAD and control groups (*P* = 0.008). There were four up-regulated microRNAs, including let-7b, miR-15a, miR-23a and US33-5p, in the AAD group compared with CP group (including 11 AMI, 2 PE and 1 AA patients; *P* = 0.000, 0.000, 0.026 and 0.011, respectively).

### MicroRNA Levels in AAD Patients

**Figure 2:**
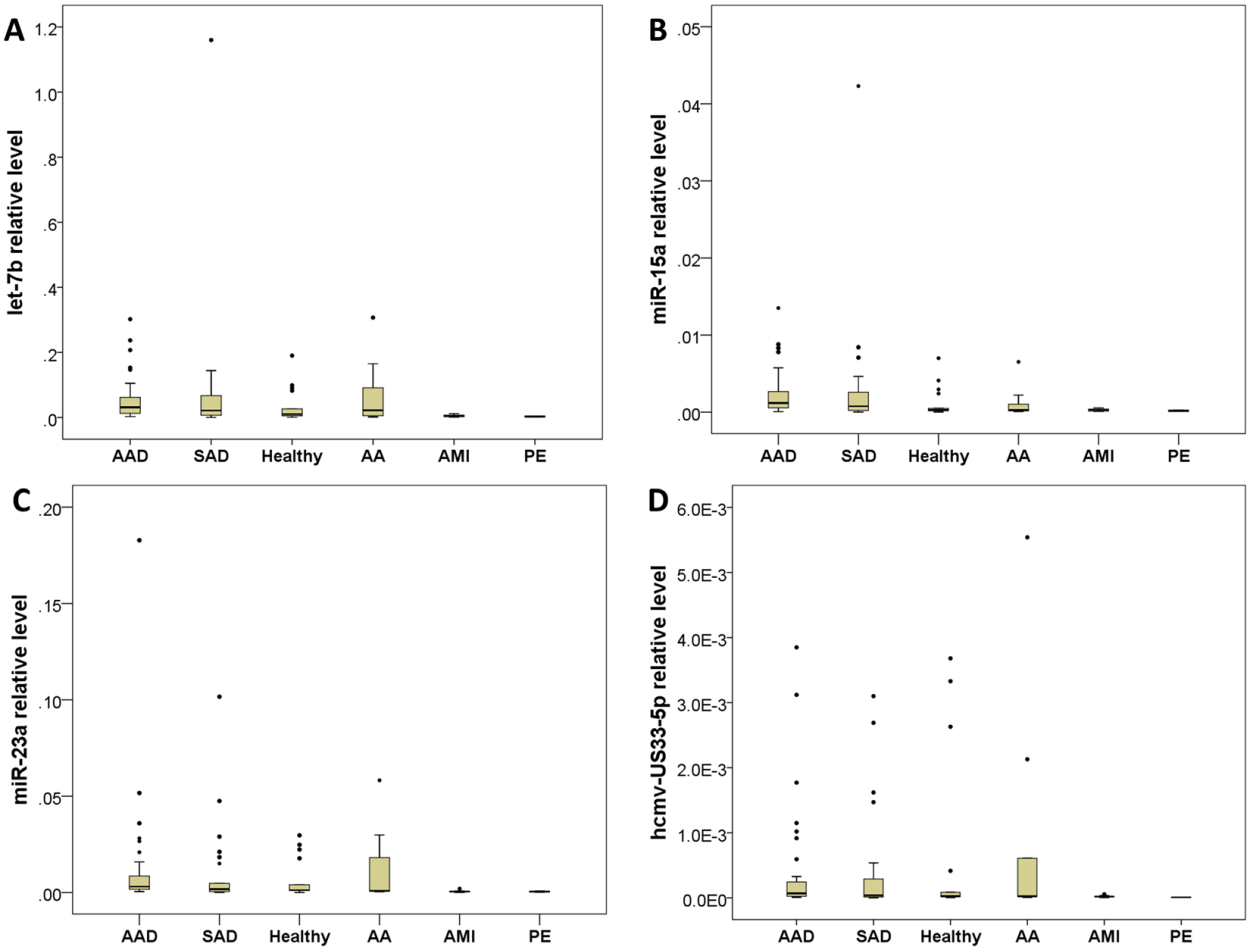
Plasma MicroRNAs Different Expression Level Between AD and Control Groups. The different expression levels of validated plasma microRNAs (including let-7b **(A)**, miR-15a **(B)**, miR-23a **(C)**, hcmv-miR-US33-5p **(D)** between AD and control groups. AAD, acute aortic dissection; SAD, subacute aortic dissection; AA, aortic aneurysm; AMI, acute myocardial infarction; PE, pulmonary embolism.

**Figure 3:**
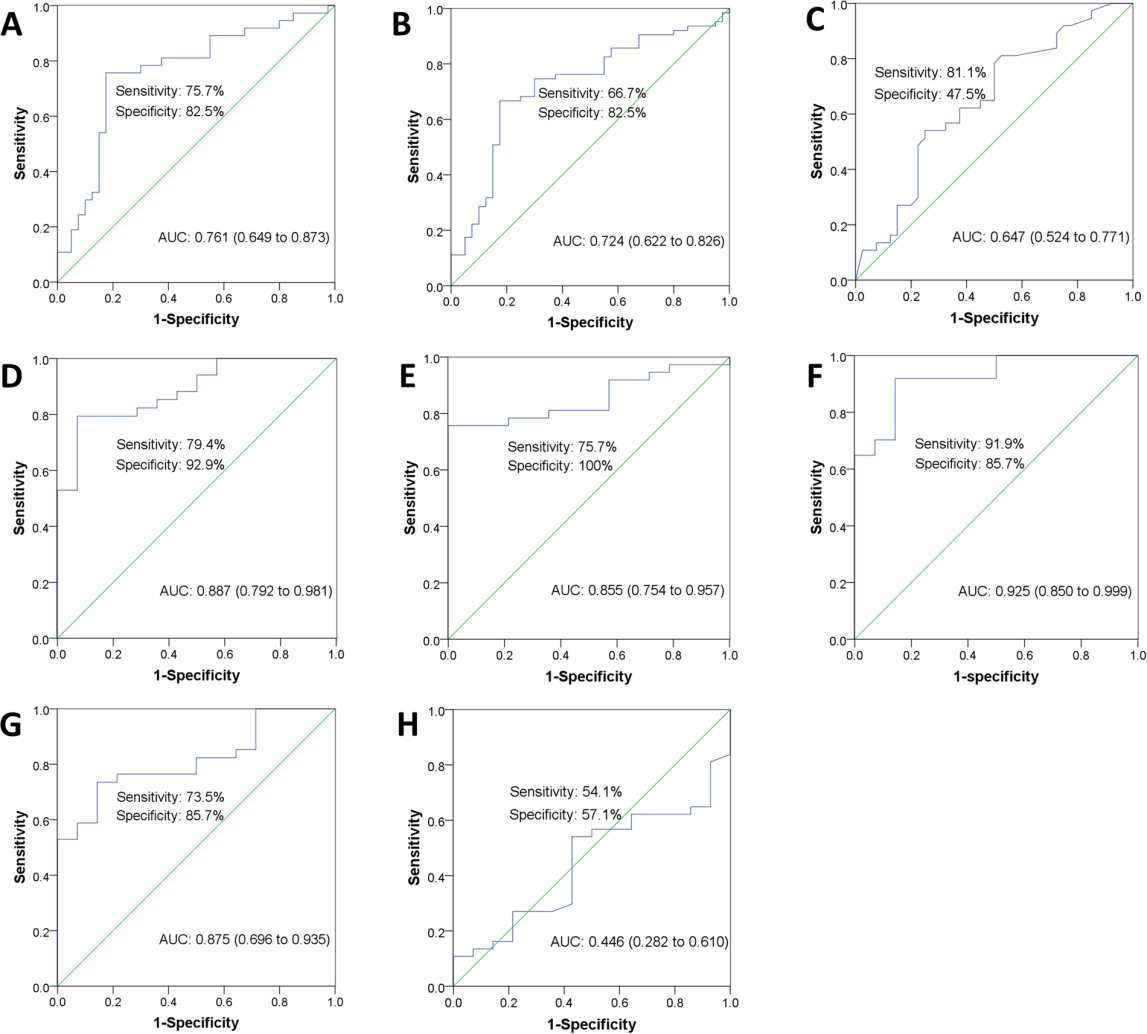
Receiver operating characteristic (ROC) curve analysis for acute aortic dissection diagnosis. Area under the curve (AUC) estimation for miR-15a in (**A**) acute aortic dissection (AAD) and control groups, (**B**) aortic dissection (including AAD and SAD) and control groups; D-dimer in (**C**) AAD and control groups. Calculation of optimal cutoff value between AAD and chest pain groups by ROC analysis with respect to let-7b (**D**), miR-15a (**E**), miR-23a (**F**), US33-5p (**G**) and D-dimer (**H**), respectively. Control groups (n = 40) includes 17 healthy participants, 11 AMI patients, 10 AA patients and 2 PE patients. Chest pain groups (n = 14) includes 11 AMI patients, 2 PE patients and 1 AA patients.

The different expression levels of validated plasma microRNAs between AD and control groups were shown in Fig. 2. The elevated level of miR-15a was observed in patients with AAD compared to the control groups (*P* = 0.008, fold change = 2.795). Furthermore, plasma level of miR-15a in AD patients was significantly higher than that in control groups (*P* = 0.016, fold change = 3.207) (Table 2). ROC analysis yielded an optimal cutoff value for miR-15a with a sensitivity of 75.7% and a specificity of 82.5% for detection of AAD; and with a sensitivity of 66.7% and specificity of 82.5% for AD detection (including AAD and SAD). The diagnostic accuracy of miR-15a measured by AUC, was 0.761 and 0.724, respectively (Fig. 3A,B; Table 2).

**Table 2:** MicroRNAs Profile and Diagnostic Performance in Training Dataset.

microRNA	AAD versus control					AAD versus CP				
	Different expression		ROC analysis			Different expression		ROC analysis		
	*P*	Fold change	sensitivity	specificity	AUC	*P*	Fold change	sensitivity	specificity	AUC
let-7b	0.126	1.747	79.4%	69.2%	0.729	<0.001	8.462	79.4%	92.9%	0.887
miR-15a	0.008	2.795	75.7%	82.5%	0.761	<0.001	8.938	75.7%	100%	0.855
miR-23a	0.213	2.123	86.5%	62.5%	0.734	0.026	17.851	91.9%	85.7%	0.925
hcmv-miR-US-33-5p	0.802	0.870	73.5%	64.1%	0.657	0.011	21.975	73.5%	85.7%	0.815
D-dimer	0.096	1.572	81.1%	47.5%	0.647	0.891	0.960	54.1%	57.1%	0.446

Control groups (n = 40) includes 17 healthy participants, 11 AMI patients, 10 AA patients and 2 PE patients. CP groups (n = 14) includes 11 AMI patients, 2 PE patients and 1 AA patients. AAD, acute aortic dissection; CP, chest pain; ROC, receiver operating characteristic; AUC, area under the receiver operating characteristic curve.

Based on clinical requirements, we further explored whether the plasma microRNA could be employed as biomarkers to distinguish between AAD and CP. When compared with CP groups, patients with AAD had significantly higher expression of let-7b, miR-15a, miR-23a and hcmv-miR-US33-5p (*P* = 0.000, 0.000, 0.026 and 0.011, respectively; fold change = 8.462, 8.938, 17.851 and 21.975, respectively; Table 2). ROC analysis for these four microRNAs exhibited sensitivity of 79.4%, 75.7%, 91.9% and 73.5%, respectively. And specificity of them were 92.9%, 100%, 85.7% and 85.7%, respectively. The corresponding AUCs were 0.887, 0.855, 0.925 and 0.815, respectively (Fig. 3D–G; Table 2). The ROC analysis exhibited microRNAs more specific than D-dimer for AAD detection in this study (Table 2; Fig. 3).

### MicroRNA Expression Profile and the Type of AAD

**Figure 4:**
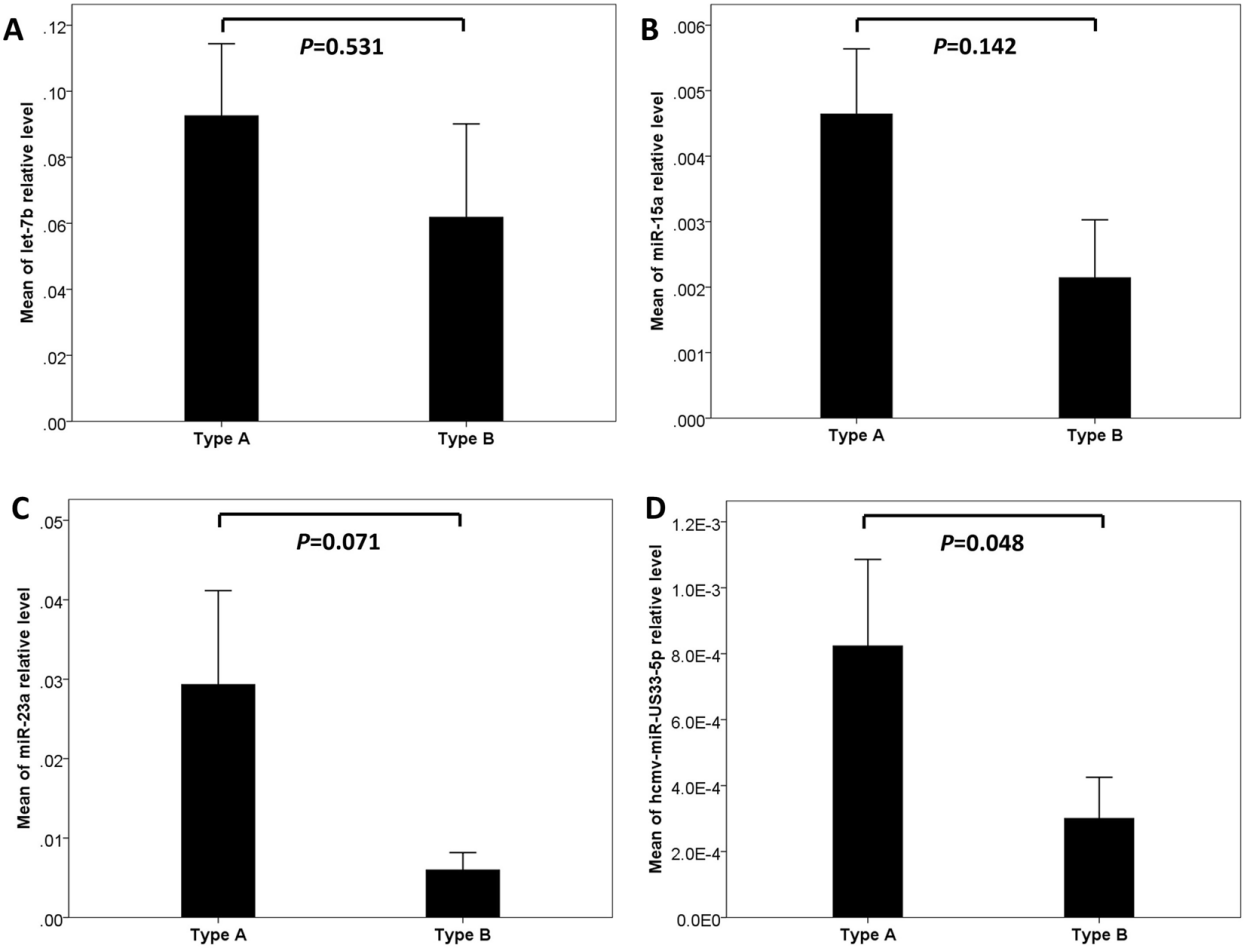
MicroRNA Expression Profile and the Type of AAD. Among the patients with AAD (Stanford type A, n = 15 and type B, n = 48), the let-7b **(A)** expression level did not exhibit any significant differences between the two types (*P* = 0.531). The levels of miR-15a **(B)** and miR-23a **(C)** tended to be higher in patients with type A AAD than in those with type B (*P* = 0.142 and 0.071, respectively). The hcmv-miR-US33-5p **(D)** level was significantly higher in patients with type A AAD than in those with type B (*P* = 0.048).

Plasma let-7b, miR-15a, miR-23a and hcmv-miR-US33-5p levels in the enrolled patients were compared according to type of dissection (Stanford type A, n = 15 and type B, n = 48). Among the patients with AAD, the hcmv-miR-US33-5p level was significantly higher in patients with type A AAD than in those with type B (*P* = 0.048). The levels of miR-15a and miR-23a tended to be higher in patients with type A AAD than in those with type B (*P* = 0.142 and 0.071, respectively). The let-7b expression level did not exhibit any significant differences between the two types (*P* = 0.531) (Fig. 4).

### MicroRNA Expression Profile and the Time from Onset

**Figure 5:**
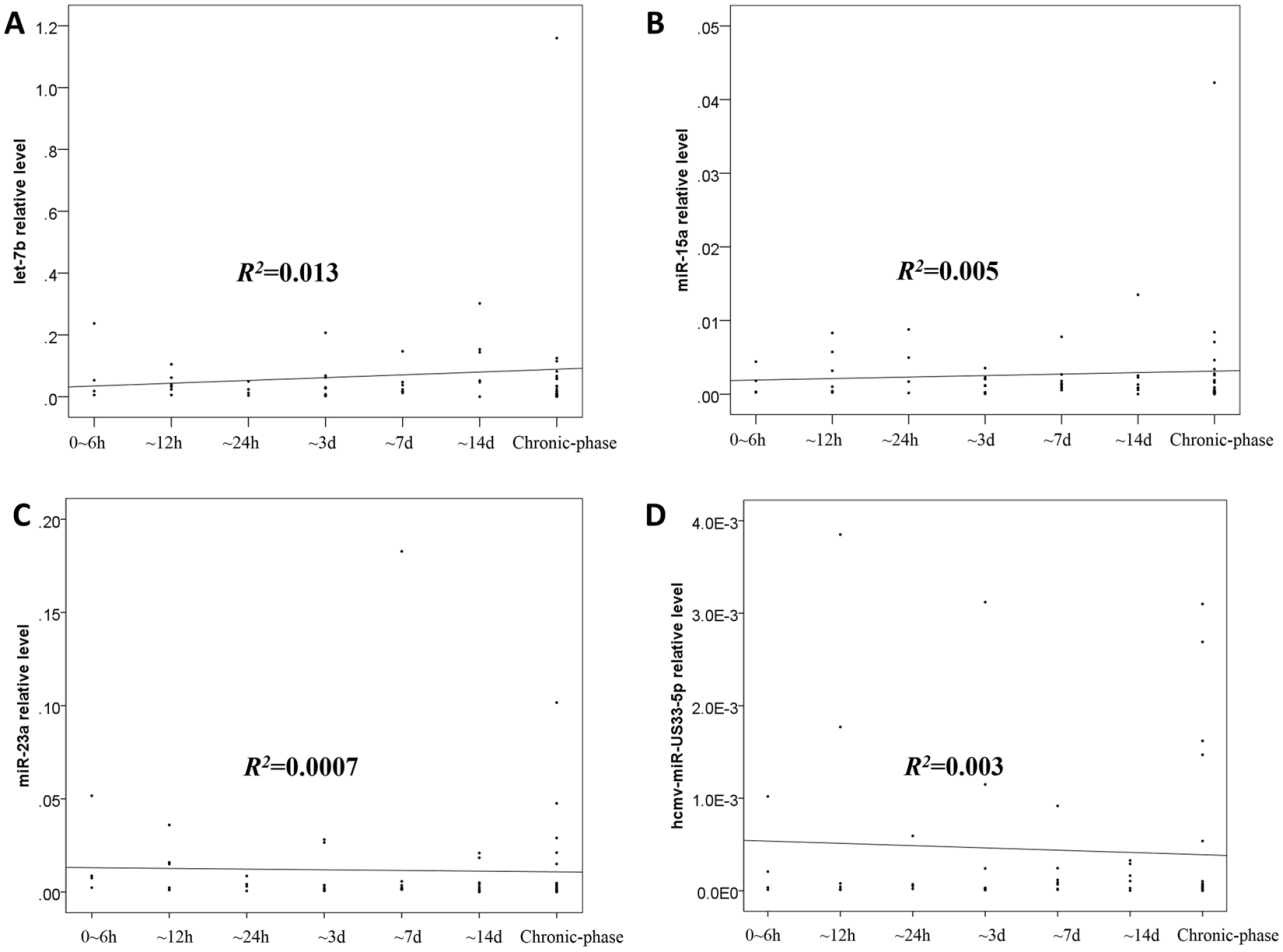
MicroRNA Expression Profile and the Time from Onset. Corelation between plasma microRNAs (including let-7b **(A)**, miR-15a **(B)**, miR-23a **(C)**, hcmv-miR-US33-5p **(D)**) and time from symptom onset in aortic dissection patients.

Further analysis of plasma let-7b, miR-15a, miR-23a and hcmv-miR-US33-5p levels in the enrolled patients according to time from onset (0~6 h, ~12 h, ~24 h, ~3d, ~7d, ~14d and subacute-phase) was shown in Fig. 5. Blood samples were collected from patients with AD on admission, and divided into 7 subgroups according to the time from onset of symptoms. By linear regression analysis, there was no correlation between plasma microRNA levels and the time from onset of symptoms (*R* ^2^ = 0.013, 0.005, 0.0007 and 0.003, respectively). This indicated that the detected microRNAs from patients with AAD were maintained at high levels accompanied by the persistence of a pathological state.

### MicroRNA Expression Profile and Outcome

**Figure 6:**
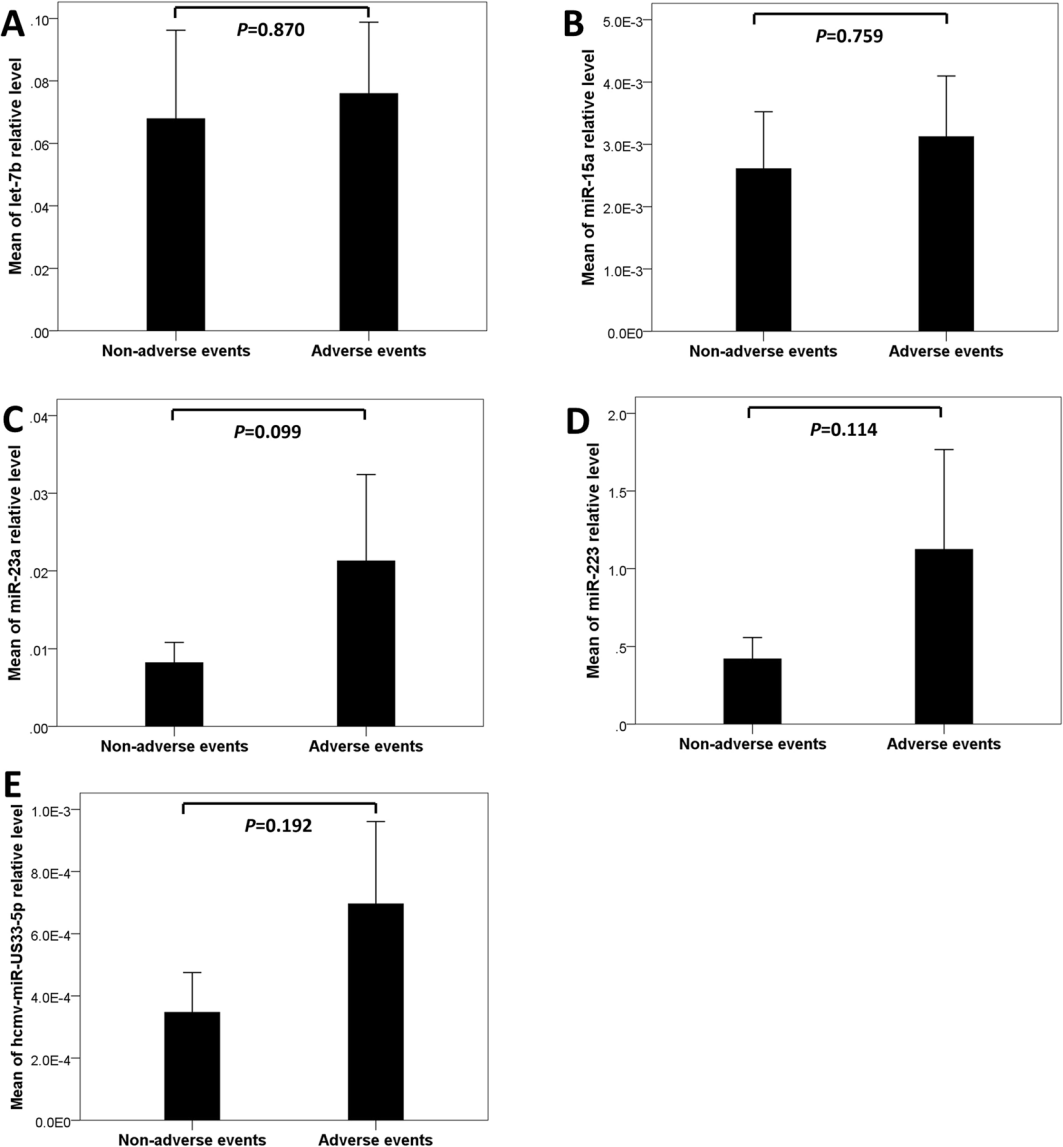
MicroRNA Expression Profile and Outcome. Adverse events included occurrence of postoperative heart failure, severe bleeding complications, postoperative intervention, bacteremia and death.

A total of four deaths (6.35%) occurred during the first 30 days. Major adverse events during postoperative course were documented in eleven patients (17.5%), including postoperative heart failure, severe bleeding complications, postoperative intervention and bacteremia. We compared the expression profiles of the microRNA in AAD patients according to their outcomes. Plasma miR-23a, miR-223 and hcmv-miR-US33-5p levels tended to be higher in patients with major adverse events than in those without major adverse events during the in-hospital period (*P* = 0.099, 0.114 and 0.192, respectively). However, the expression levels of let-7b and miR-15a did not show obvious association with major adverse events (*P* = 0.870 and 0.759, respectively) (Fig. 6).

**Figure 7:**
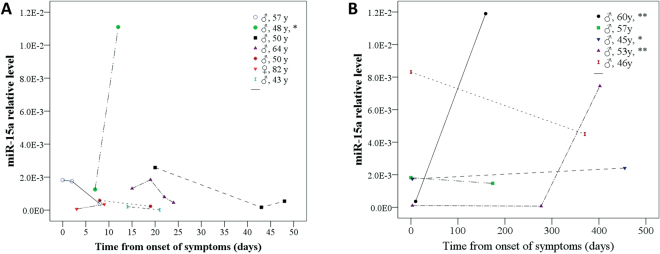
Serial Measurements of Plasma miR-15a. **(A)** Serial plasma miR-15a measurements during hospitalization of 7 AAD patients. In the 6 patients with no major adverse events, serial miR-15a measurements demonstrate a decline over time, while in one patient who occurrence of bacteremia (♂, 48 y, *), sharp increase was found. **(B)** Serial plasma miR-15a measurements during follow-up of 5 AAD patients. 2 of 5 patients with postoperative condition were stable. The plasma level of miR-15a was found to be lower than their respective plasma levels during the onset of AAD. **2 patients with recurrent AAD, their plasma miR-15a level were increased significantly. *One patient with occasional fever, his miR-15a level was sustained at a higher level and slightly higher than in-hospital.

Serial plasma miR-15a measurements during hospitalization of seven patients with AAD were presented in Fig. 7A. All of them underwent emergency endovascular repair. In the six patients without major adverse events, serial miR-15a measurements demonstrated a decline over time, while in one patient with occurrence of bacteremia a sharp increase was observed.

A follow-up was performed to further determine whether plasma miR-15a in AAD patients presented any changes after endovascular repair (Fig. 7B). The blood was collected from five patients with AAD for serial miR-15a detection. Two of the five patients with postoperative conditions were stable. The plasma level of miR-15a was found to be lower than their respective plasma levels during the onset of AAD. Two patients with recurrent AAD experienced significantly increased levels of plasma miR-15a. One patient experienced occasional fevers, sustained a high level of plasma miR-15a which was slightly higher than in-hospital.

### Characterization of Plasma microRNA Stability

**Figure 8:**
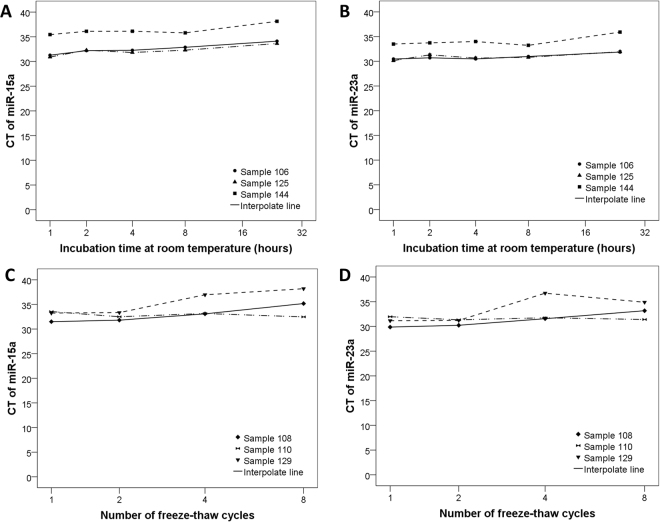
Characterization of Plasma microRNA Stability. MicroRNAs levels remain stable when plasma is subjected to prolonged room temperature incubation (**A** and **B**) or freeze-thawed multiple times (**C** and **D**).

The expression stability was an important prerequisite for plasma miRNAs serving as a biomarker. In the current, we investigated the stability of microRNAs in plasma. Incubating the plasma at room temperature for up to 24 hours (Fig. 8A,B) and subjecting it to up to four cycles of freeze-thawing (Fig. 8C,D) had minimal effect on miR-15a and miR-23a levels as measured by qRT-PCR.

We sought to determine if microRNA measurements were substantially different between plasma and serum, by measuring the miR-15a and miR-23a in matched samples of plasma and serum collected from a participant at the same blood draw. From the measurements obtained there was no discernible difference between either samples.

## Discussion

At the present time, the diagnosis of AAD is based on clinical presentation and mainly relies on imaging techniques. However, the diagnostic performance of these modalities is unsatisfactory for the diagnosis of AAD. At this time, up to 39% of patients remain undiagnosed until necropsy^9^. A diagnostic biomarker for AAD will reduce delays in diagnosis and promote the early diagnosis rate, potentially improving the prognosis of patients. Until now, there are lack of a biomarker with high enough sensitivity and specificity, nor with a long enough time-window for positively identifying AAD. The recent discovery of aberrant expression of microRNAs in aortic tissue from AAD patients has paved the way for analyzing circulating microRNAs for the purpose of AAD diagnosis.

Blood-based biomarker test is convenient, low cost, fast and effective that may be a promising approach for routine screening and surveillance of AAD. In this study, we found that circulating microRNAs were significantly altered in AAD. In particular, our study revealed that patients with AAD had significantly higher expression levels of let-7b, miR-15a, miR-23a and hcmv-miR-US33-5p when compared with CP groups. Among them, miR-23a exhibited the highest diagnostic accuracy for AAD. When compared with control groups, patients with AAD had significantly higher expression levels of miR-15a, reveling its good diagnostic accuracy for detection of both AAD and SAD. Then, plasma miR-23a might have considerable clinical value for the initial diagnostic work-up of patients with CP and suspected AAD. Plasma miR-15a was important in monitoring the progression of and screening for AAD from the varied presentation, including asymptomatic patients.

Previous studies suggested a number of candidate biomarkers for AAD. Determination of smooth muscle myosin heavy chains and soluble elastin fragments showed high sensitivity and specificity in diagnosis of AAD, but these tests were not practical in a clinical emergency setting. Experience with calponin was limited, further investigation was required to improve sensitivity and specificity. D-dimer levels exhibited an excellent sensitivity for the detection of AAD, but had a lower specificity (46.6% to 68.6%)^23–26^. And subacute aortic dissection will be missed as a result of the short biological half-life of D-dimer^26^. Interestingly, circulating microRNAs showed higher diagnostic accuracy and a longer time-window for diagnosis of AAD in this study.

In previous studies, the control group forms of healthy volunteers and cases with similar symptoms of AAD, which was undoubtedly the primary factor to consider. However, there was a similar organizational structure in AA, along with similar risk factors and a series of hemodynamic changes, which might be applied as improved biomarkers in diagnosing aortic dissection. In this study, AA was used as a case control in the process of screening and validating differentially expressed circulating microRNAs in AAD. We believed that the results obtained in this way was more specific and reliable.

In earlier studies on cardiovascular diseases, microRNAs have also been proposed to possible be used for their diagnosis and prognosis as well. For example, miR-23a was found to be elevated in patients with coronary artery disease, which suggested that it might served as a biomarker for the disease development^27^; while miR-23 was reported to be enriched in the plasma of CAD cases, implying its potential function as a diagnostic tool^28^. All these evidences along with our findings supported the promising role of microRNAs in diagnosing cardiovascular diseases. In our study, when compared with CP groups (includes 11 AMI patients, 2 PE patients and 1 AA patients), patients with AAD had significantly higher expression of miR-23a (P = 0.026; fold change = 17.851). Discriminating between AAD and AMI provides important therapeutic implications. Recent reports have led us to identify a set of microRNAs that can be clinically practicable biomarkers for AMI diagnosis. Our present work also suggested that microRNAs in plasma might be sensitive and specific biomarkers for AAD. Circulating microRNAs may provide a helpful and reliable tool in the diagnosis of AAD or AMI.

### Study limitations

The outcome of the patients in this study comes from in-hospital observations and short-term follow-ups, limiting our current ability for prognostic analysis. The data in our study from a single center study has lower dispersion. Additionally, the sample size is relatively small in the current study. Therefore, confirmation in multicenters studies with larger sample size is still necessary.

## Conclusion

In summary, plasma miR-15a levels are significantly different between AAD and control groups (including healthy controls, AMI, AA and PE). And plasma miR-23a has a high sensitivity and specificity for differentiating AAD from CP groups. They have a long time-window and high degree of accuracy for detecting AAD. Our study demonstrates that plasma miR-23a exhibits considerable clinical value for the differential diagnosis of AAD. Plasma miR-15a can be used in monitoring and screening of AAD. The newly identified biomarkers may promote the early diagnosis rate of AAD, thus improving the prognosis of the patients. So that more patients with acute chest pain can benefit from the optimal therapy who would have otherwise missed the curative treatment window.
